# Optimized Injectable Matrix Approach for Deep Margin Elevation in Challenging Subgingival Restorations: A Case Report

**DOI:** 10.1155/crid/1221045

**Published:** 2025-11-25

**Authors:** Ahmad Toumaj, Sadaf Akbarikajani, Mahtab Mottaghi

**Affiliations:** ^1^Department of Prosthodontics, Faculty of Dentistry, Islamic Azad University Tehran Medical Sciences, Tehran, Iran; ^2^School of Dentistry, Babol University of Medical Sciences, Babol, Mazandaran, Iran; ^3^School of Dentistry, Mashhad University of Medical Sciences, Mashhad, Iran

**Keywords:** custom matrix, deep margin elevation, subgingival restoration, tissue management

## Abstract

This case report describes a modified deep margin elevation (DME) technique to address a deep subgingival carious lesion in Tooth 9 of a 62-year-old female patient with a history of bisphosphonate therapy and hypothyroidism. Conventional restorative techniques were inadequate because of the lesion's subgingival depth. A customized injectable matrix technique was utilized, incorporating injectable wax, flowable composite, and soft tissue management with aluminum chloride–soaked retraction cord. The wax replicated the internal structure of the cavity, upon which a flowable composite was light-cured to form a patient-specific matrix. Glycerin gel was used to eliminate the oxygen-inhibited layer and facilitate matrix clearance. Composite resin was administered to raise the margin supragingivally, enhancing both periodontal and restorative results. This technique provides superior accuracy, clarity, and flexibility compared to conventional DME, particularly in anatomically complex cases.

## 1. Introduction

Restoring teeth with severe subgingival cavities is a major clinical challenge for dental professionals [1]. When carious lesions progress beyond the cementoenamel junction (CEJ) and the cavity edges are located beneath the gingival tissues, doctors face complex therapeutic difficulties [2, 3]. Common challenges encompass the thorough removal of caries, cavity preparation, impression-taking, effective isolation, accurate application of restorative materials, elimination of cement excess, and avoidance of supracrestal tissue attachment (SCTA) infringement—all of which are impacted by deep cervical lesions. Contamination from saliva, blood, or gingival crevicular fluid can adversely affect adhesion and marginal integrity, hence diminishing the durability of restorations [4].

Subgingival margins and overhanging restorations are linked to periodontal complications, such as enhanced bleeding on probing, increased probing depths, loss of clinical attachment, and radiographic bone loss. Treating subgingival carious lesions presents significant clinical challenges, particularly regarding moisture control and contamination prevention [3].

Various therapeutic options have been suggested for managing these cases, including orthodontic extrusion, surgical crown lengthening, deep marginal elevation (DME), and extraction [5–8]. Surgical crown lengthening may adversely affect alveolar bone height in the treated region and adjacent teeth, potentially resulting in unfavorable crown-to-root ratios and root exposure. Orthodontic extrusion is effective but requires considerable time and is affected by anatomical and biomechanical factors [6]. Surgical crown lengthening may result in complications including furcation exposure, violation of the biological width, postoperative discomfort, and decreased patient acceptance [9]. Extraction is likely not a feasible option for patients on bisphosphonates because of the associated risk of bisphosphonate-related osteonecrosis of the jaw (BRONJ) [10–12].

Deep cervical cavities frequently lead to two primary complications: biological and technical issues [2]. Restricted visibility and access to deep cavity regions, coupled with insufficient rubber dam isolation, complicate moisture control and elevate the risk of contamination from saliva and blood during the procedure [2]. Ensuring sufficient isolation in deep approximal lesions, especially for enamel bonding, presents a considerable challenge [13, 14].

DME, introduced in 1998, is a minimally invasive restorative technique known as cervical margin relocation, proximal box elevation, or coronal margin relocation [15, 16]. DME is aimed at repositioning deep subgingival margins to a supragingival location through using various restorative materials, including conventional composite resins, flowable composites, bulk-fill composites, glass ionomer cements, and resin-modified glass ionomers [1, 17, 18]. This technique minimizes treatment duration and the number of appointments, maintains the biological width [19, 20], and has shown significant clinical success, even in instances where the defect approaches the biological width [6, 21–23]. Research indicates that DME provides superior restoration longevity and reduced inflammation when compared to surgical crown lengthening [4, 22, 24].

This study presents a technique that employs a custom-designed tray for DME application across all tooth surfaces, compared with prior methods that predominantly targeted interproximal areas using steel or plastic matrix bands. The process was applied to the lingual surface. This novel technique provides an alternative to surgical crown lengthening, orthodontic extrusion, or tooth extraction in specific cases and can be performed in a single session. The method facilitates the formation of a clean, dry composite build-up in teeth with subgingival margins while avoiding negative periodontal reactions resulting from SCTA violation. The approach intends to provide substantial socioeconomic advantages.

## 2. Case Presentation

**Figure 1:**
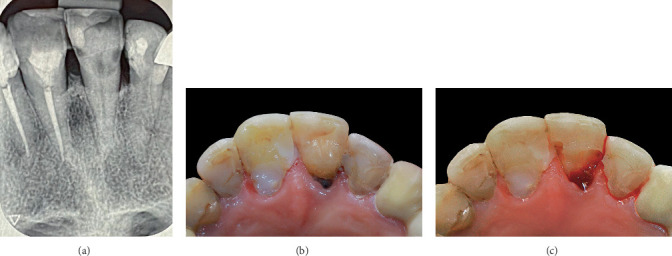
(a) Periapical radiograph of maxillary anterior teeth. (b) Initial image depicting the palatal surface of the maxillary anterior teeth. (c) Tooth 9 after caries removal.

A 62-year-old female with a history of bisphosphonate medication for osteoporosis for over 4 years and hypothyroidism was referred to a private dental office for a routine checkup. Clinical examination and preapical radiograph of the anterior maxilla showed caries in Tooth 9. The teeth were vital, and no periapical pathology could be seen on radiographic assessment (Figure 1a). Due to the lesion's subgingival extent in Tooth 9 (Figure 1b), the conventional restorative method was considered unsuitable. A modified version of the DME procedure was utilized, integrating a custom-designed injectable matrix technology with a soft tissue separation strategy to enhance results in deep subgingival restorations.

### 2.1. Atraumatic Caries Removal and Isolation Without Rubber Dam

Following an injection of local anesthetic, all infected dentin was meticulously removed (Figure 1c). According to clinical and radiographic assessment, endodontic treatment was not indicated. The tooth was asymptomatic and had no radiological evidence of periapical disease (Figure 1a). Despite the significant depth of the lesion, no pulpal exposure was noted, supporting a cautious restorative strategy. Due to the subgingival depth of the lesion (about 3.5 mm), conventional rubber dam isolation, even with a #212 retraction clamp, proved impractical. The lesion depth and its proximity to the alveolar crest were meticulously evaluated to avoid infringing upon SCTA. Controlled gingival displacement was achieved utilizing a retraction cord and Teflon tape to preserve biological width while facilitating successful isolation. This method facilitated moisture regulation and enhanced visibility in a typically inaccessible surgical area. The region was disinfected with an antiseptic mouth rinse and treated with isotonic saline.

### 2.2. Controlled Gingival Displacement for Matrix Impression

**Figure 2:**
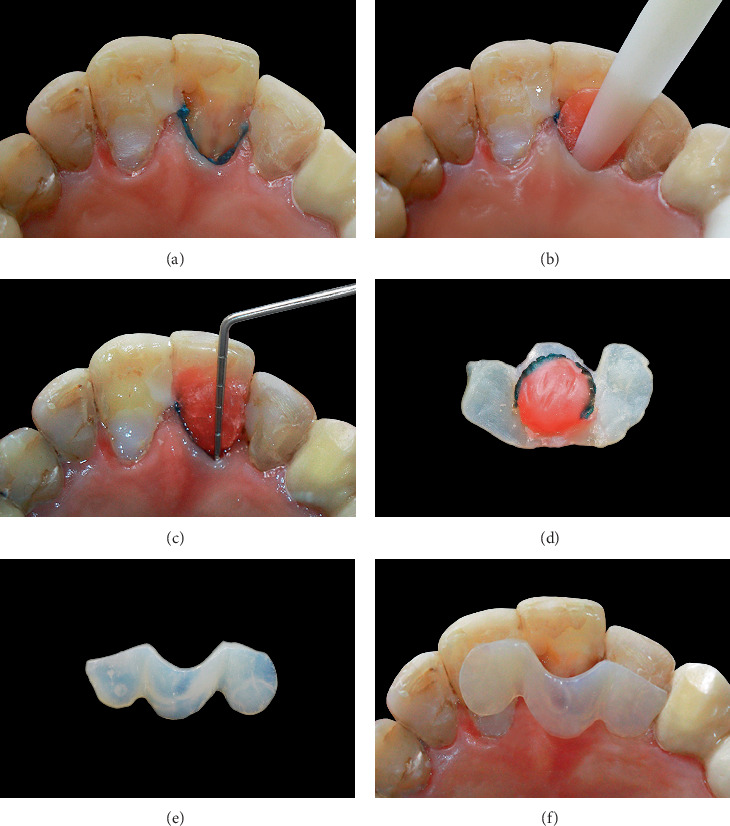
(a) Inserting a hemostatic solution–soaked retraction cord into the sulcus. (b) Application and compaction of injectable wax to ensure thorough adaptation to the enamel cavity. (c) The wax material extends over 3.5 mm to fully cover the margins of the enamel cavity. (d) The composite matrix, embedded wax, and retraction chord after removal. (e) The final custom-made matrix. (f) Custom-made matrix positioned on the teeth.

A retraction cord soaked with 25% aluminum chloride hemostatic solution (Size 1) was delicately inserted into the sulcus of Tooth 9 (Figure 2a). This step facilitates tissue displacement in the next steps, reducing damage and allowing for precise subgingival capture.

### 2.3. Internal Cavity Impression Using Sculptable Wax

The internal surfaces of the cavity were disinfected with 2% chlorhexidine. The warmed wax was inserted into the cavity in a small ball and meticulously adapted with a plastic instrument to ensure full contact with all internal surfaces. Incremental adaptation and gentle compaction of the softened wax were conducted under magnification to attain a comprehensive peripheral seal without any voids, hence ensuring precise replication of the cavity structure. The wax was adapted to extend 3.5 mm coronally from the cavity margin (Figure 2b). Excess material was removed to eliminate overextensions. The wax extends over 3.5 mm (Figure 2c) to fully cover the cavity margins. This phase represents a crucial progression in customized matrix fabrication, as it precisely defines the undercut and subgingival morphology necessary for effective repair.

### 2.4. Resin-Based Custom Matrix Design

A flowable composite resin was incrementally applied over the wax, allowing for precise control in the sulcular region. The composite was extended to neighboring teeth and light-cured to create a rigid customized matrix. The layer thickness was kept at 1–1.5 mm to guarantee sufficient rigidity and precise seating without deformation. The composite slightly extended into the sulcus to ensure a future gingival seal during restoration—a design rarely accomplished by prefabricated matrices. The composite matrix, embedded wax, and retraction cord were carefully extracted as one unit. This procedure enabled the production of a patient-specific matrix that could be accurately adjusted during the final restoration (Figure 2d). The excess composite was removed.  Figure 2e shows the final custom-made matrix.

### 2.5. Sulcus Isolation and Enamel Preparation

A retraction cord soaked with 25% aluminum chloride hemostatic solution was placed in the sulcus. After 5 min, the area was meticulously rinsed with normal saline solution. The enamel borders were beveled, and selective etching was conducted with phosphoric acid. A self-etch adhesive was utilized and light-cured according to the manufacturer's instructions.

### 2.6. Glycerin-Coated Matrix Repositioning

The customized matrix was repositioned, and its fit was confirmed both visually and tactually to ensure full adaptation to the palatal and proximal margins (Figure 2f). Stability during resin injection was achieved by securing the matrix to adjacent teeth using small dots of flowable composite, applied without etching or bonding. The preformed wax pattern facilitated accurate placement even with the smooth palatal surface. Before resin injection, a thin layer of glycerin gel was placed on the inside surface of the matrix. This served two purposes:

• To prevent the adhesion between the matrix and the restoration, enabling uncomplicated removal.
• Eradicating the oxygen-inhibited layer enhances surface polymerization and ensures prolonged color and mechanical stability.

### 2.7. Restoring Process

**Figure 3:**
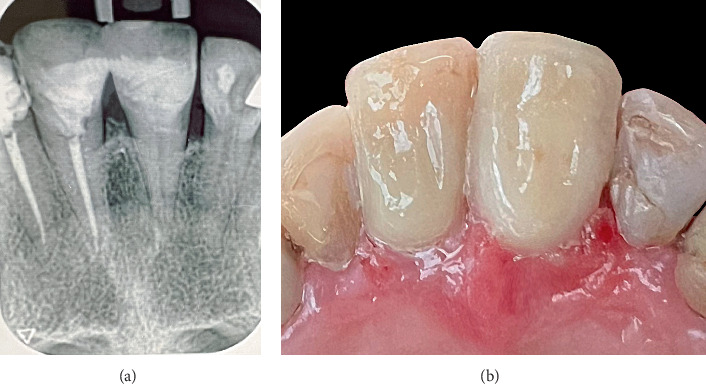
(a) Periapical radiograph of the final restoration. (b) Clinical view of the completed restoration.

The flowable composite resin was incrementally injected through the palatal access obtained by the lesion's extension, eliminating the necessity for creating a separate injection hole. The lesion's depth limited direct visualization; thus, adaptation was confirmed via tactile confirmation and the controlled emergence of resin at the coronal margin. Each increment was injected slowly under constant pressure to ensure complete filling of the cavity and proper marginal adaptation, thereby converting the deep subgingival lesion into a supragingival one with both periodontal and restorative benefits. A thin layer (approximately 0.5 mm) of flowable composite resin was initially injected through the palatal access with the customized matrix in place. The initial increment was light-cured for 30 s to raise the restoration margin above the gingival level. The matrix was then removed, and the residual cavity was restored with subsequent increments of composite, each light-cured for 30 s utilizing a high-intensity LED light curing unit (Bluephase PowerCure, Ivoclar-Vivadent). This method achieved sufficient polymerization and optimal marginal adaptation and maintained mechanical integrity at a subgingival depth of 3.5 mm. Final finishing and polishing were conducted to guarantee an appropriate emerging profile and marginal adaptation. Additionally, due to the color alteration of the composite restoration in the adjacent tooth (Tooth Number 8), the composite restoration was removed and restored.  Figure 3 shows the final periapical radiograph and clinical view of the teeth.

## 3. Discussion

The dental clinician has traditionally been cautious about restoring deep subgingival lesions due to their frequent association with substantial defects exhibiting subgingival margins that extend beyond the CEJ [25]. Subgingival preparations provide challenges that can affect subsequent procedures, including rubber dam isolation, digital and conventional impression taking, restorative implantation, cementation, and finishing and polishing of the cervical region. Furthermore, indirect partial posterior restorations frequently exhibit subgingival edges, which are associated with both biological and operational complications [4]. Lesion extending beyond 3 mm subgingivally may risk biologic width violation; hence, minimally invasive approaches such as DME and controlled gingival displacement were adopted in this case.

The introduction of the DME technique offers several advantages, such as effective isolation. Recent investigations have further clarified the biomechanical and biological implications of various DME materials and techniques, including improved moisture control, enhanced impression-taking, optimized bonding procedures, and the reduction of excess material while minimizing unnecessary tissue removal [15]. Despite all the advantages, the success rate of conventional DME is frequently limited by anatomical variations, inadequate isolation, and challenges in matrix placement, which may not accurately fit complex subgingival contours [16].

This report presents a modification to the conventional DME technique through introducing a custom-made injectable matrix system. This system is created through intraoral wax molding, followed by the addition of flowable composite resin. The patient-specific matrix accurately captures the subgingival contours of the lesion, provides a precise seal, and includes an occlusal injection channel for controlled composite delivery. In contrast to prefabricated matrixes, this approach allows for superior adaptation in anatomically challenging cases, ensuring predictable elevation of deep margins with enhanced precision and marginal integrity.

Recent studies have revealed the biomechanical and biological effects of various DME materials and techniques. Salah and Sleibi [26] conducted a study examining the impact of elevating margins at 2 and 3 mm below the CEJ using either bulk-fill flowable composite or short fiber–reinforced flowable composite (SFC). The findings demonstrated that the degree of margin elevation (2 mm compared to 3 mm) did not have a statistically significant impact on fracture resistance. The application of flowable composite within a specific injectable matrix may replicate comparable mechanical benefits by enhancing internal stress distribution and restorative contour.

The application of flowable composite through injection to restore contour and shape has been explained in previous studies [27]. A flowable composite functions as a stress-absorbing layer beneath the filled hybrid composite resin repair. This might be explained by the notion of an “elastic wall,” which depends on the low elastic modulus and the high wettability of flowable materials, allowing them to serve as an intermediate layer [4]. Consistent with these findings, our suggested method employs injectable flowable composite to improve marginal adaption and internal stress distribution in deep subgingival restorations, therefore reinforcing both mechanical integrity and periodontal compatibility.

However, biological factors are essential in subgingival repairs. A recent clinical by Hausdörfer et al. [28] evaluating periodontal outcomes of proximal DME combined with lithium disilicate partial restorations indicated a significantly elevated incidence of BOP in DME sites relative to control sites after 1 year. Despite steady probing depths over time, the rise in BOP indicates heightened gingival inflammation at DME locations, especially when edges are positioned below the gingival sulcus. The increase in plaque index across all surfaces highlights the importance of meticulous hygiene practices and careful material selection. The data suggest that whereas DME enhances restorative effectiveness and fracture resistance, it may elevate the risk of periodontal irritation if soft tissue management is insufficient.

In the present case report, the application of Teflon tape and retraction cord for gentle soft tissue displacement, combined with antiseptic cleaning and glycerin coating, was aimed at minimizing inflammatory responses while facilitating appropriate margin elevation. Rubber dam isolation was found impractical due to the 3.5-mm subgingival extension of the lesion and the limited accessibility of the anterior palatal region. Despite using a #212 retraction clamp, achieving an adequate seal was not possible without the risk of tissue damage. Soft tissue management was accomplished through the use of retraction cord and Teflon tape to ensure moisture control and visibility. This conservative approach reduced trauma and was especially appropriate for a patient receiving long-term bisphosphonate therapy, where excessive tissue manipulation was contraindicated.

The current case illustrates the potential benefits of a customized matrix-guided DME approach, although many limitations must be recognized. The report relies on a single clinical case, which limits its capacity to draw generalized conclusions. The absence of comparative analysis with ordinary matrix systems limits the evaluation of its relative superiority. The periodontal reaction to subgingival matrix insertion is a concern, as inflammatory alterations may develop over time, particularly in those with inadequate oral hygiene or weakened immunity. Moreover, the procedure requires operator proficiency and additional chairside time, which may not be feasible in all clinical environments. Controlled clinical trials and defined methods are necessary to confirm the reproducibility, efficacy, and long-term results of this method.

This technique enables dentists to efficiently restore teeth with extensive lesions while conserving dental structure and minimizing invasive procedures. It addresses operability with biological integrity, providing a feasible alternative to more invasive surgical or prosthodontic procedures. However, longitudinal data are necessary to assess if customized matrix-guided DME can reliably alleviate the physiologic disadvantages associated with conventional DME.

In conclusion, the restoration of deep subgingival lesions presents a complex clinical challenge due to anatomical constraints and biological risks. The modified DME technique, incorporating a custom-designed injectable matrix, strategies for soft tissue preservation, and the application of a flowable composite, exhibits a promising method for achieving optimal restorative and periodontal results in anatomically challenging cases. By enhancing margin adaptation, facilitating moisture control, and maintaining gingival integrity, this technique may improve the predictability of subgingival restorations. Nonetheless, its successful application requires meticulous case selection, operator expertise, and strict adherence to tissue management protocols. Future clinical investigations with larger sample sizes and extended follow-up are crucial to substantiate the efficacy and safety of this approach in routine dental practice.

## Data Availability

The data that support the findings of this study are available from the corresponding author upon reasonable request.
